# Satisfied and Secured—An Integration of Self-Determination Theory and Attachment Theory in the Environmental Domain

**DOI:** 10.3390/ejihpe15040062

**Published:** 2025-04-15

**Authors:** Jean-François Bureau, Ariane J. Gauthier, Shanna With, Audrey-Ann Deneault, Nicole Racine, Simon G. Beaudry, Steve Lorteau, Luc G. Pelletier

**Affiliations:** 1 School of Psychology, University of Ottawa, 136 Jean-Jacques-Lussier Private, Ottawa, ON K1N 9A8, Canada; agaut041@uottawa.ca (A.J.G.); swith077@uottawa.ca (S.W.); nracine2@uottawa.ca (N.R.); simon.beaudry@uqo.ca (S.G.B.); luc.pelletier@uottawa.ca (L.G.P.); 2 Data Literacy Research Institute, University of Ottawa, 60 University Private, Ottawa, ON K1N 8Z4, Canada; 3 Département de Psychologie, Université de Montréal, 90 Vincent-d’Indy Avenue, Montreal, QC H2V 2S9, Canada; audrey-ann.deneault@umontreal.ca; 4 Décanat de la Recherche et de la Création, Université du Québec en Outaouais, 283 Alexandre-Taché Boulevard, Gatineau, QC J8X 3X7, Canada; 5 Faculty of Law, University of Ottawa, Louis-Pasteur Private, Ottawa, ON K1N 8Z4, Canada; steve.lorteau@uottawa.ca

**Keywords:** self-determination theory, attachment theory, pro-environmental behaviors, climate change, environmental motivation

## Abstract

While environmental motivation research has investigated several factors that can facilitate and promote the adoption of pro-environmental behaviors, questions remain on how individuals can be brought to change their behaviors and habits. In the current study, we draw on attachment theory and self-determination theory to better understand why motivational interventions meant to increase pro-environmental behaviors are ineffective for some individuals. Using a person-centered approach, our analysis uncovered four latent profiles characterized by varying levels of attachment insecurity and basic psychological need satisfaction. Further analysis suggests that these four profiles are associated with distinct motivational pathways in the environmental domain. Our results suggest that self-determined motivation is a direct predictor of pro-environmental behaviors solely for individuals from the secure attachment and high-need satisfaction profile. This association was not observed in individuals arising from insecure attachment and low-need satisfaction profiles, suggesting that the association between motivation and pro-environmental behaviors commonly reported in the literature might be moderated by one’s social environment. Implications for motivation researchers and policymakers are discussed, such as the relevance of considering attachment when designing motivational interventions in the environmental domain.

## 1. Introduction

Climate change and its attendant disasters represent a direct and serious threat to humanity in terms of economic, health, and social consequences (Intergovernmental Panel on Climate Change, 2023). Facing the spectre of major environmental change, many young people report experiences of climate-related distress (Hickman et al., 2021; Ojala et al., 2021), an umbrella term referring to the negative emotions and mental health problems caused by their perception of climate change, such as anxiety, sadness, and guilt (Feather & Williams, 2022). The awareness of climate change can prompt different responses, ranging from paralysis to climate inaction (Masud et al., 2015; Verplanken et al., 2020). These diverse reactions have important consequences for how we address climate change, underscoring the need to understand the motivational precursors of pro-environmental behaviors (PEBs; Lavergne & Pelletier, 2015).

Using Self-Determination Theory (SDT; Ryan & Deci, 2017), past research has extensively examined the role of motivation in fostering PEBs. However, this literature cannot fully explain why individuals presenting strong environmental attitudes and feeling motivated do not engage in PEB (Carrington et al., 2014; Farjam et al., 2019; Lavergne & Pelletier, 2015; van Valkengoed et al., 2022). In the current study, we examine if attachment theory (Bowlby, 1969), a theory that examines how the social environment can impact one’s ability to cope in the face of threats, could help fill this gap in the literature. By simultaneously considering attachment theory and SDT, this study seeks to deepen our understanding of intra-individual factors that could influence how environmental attitudes are translated into PEB.

### 1.1. Self-Determination Theory

Self-determination theory (Ryan & Deci, 2000) is a leading theory of human motivation that has been applied to a wide range of domains, including the environmental domain (Pelletier et al., 1998, 2011). This theory helps explain how behaviors are initiated and maintained (Ryan & Deci, 2017), as well as the impact of the social environment and relationships on personal growth (La Guardia & Patrick, 2008). The concept of motivational quality is central to SDT. The theory suggests that the motives underpinning one’s behaviors vary in their degree of internalization, which is dependent on the environment of the individual (Ryan & Deci, 2017). The reasons for which individuals engage in different behaviors are described along a continuum of internalization ranging from the least self-determined (amotivation) to the most self-determined type of motivation (intrinsic motivation). Under SDT, humans are naturally motivated, curious, and agentic and pursue connections with others (La Guardia & Patrick, 2008; Whipple et al., 2009). However, this tendency depends on context, as social interactions can also influence one’s motivation and well-being (Deci & Ryan, 1985). One way through which the social environment influences one’s motivational regulation is through the satisfaction or frustration of basic psychological needs (Sheldon, 2011).

#### 1.1.1. Basic Psychological Needs Theory

According to SDT, humans have three basic psychological needs (autonomy, competence, and relatedness) that must be satisfied in order for them to thrive (Deci & Ryan, 2002; Ryan & Deci, 2000). These three needs are assumed to be universal and innate, although the ways through which they are satisfied may vary from one culture to another or from one developmental stage to another (Deci & Ryan, 2002). The need for autonomy refers to one’s feelings of willingness and volition regarding their behaviors (Ryan & Deci, 2017). The need for competence refers to one’s need for opportunities to expand and demonstrate their skills and abilities (Ryan & Deci, 2017). Lastly, the need for relatedness refers to one’s need to feel connected with others, to care, and to be cared about (Ryan & Deci, 2017). These needs can be supported or thwarted by the individual’s social environment (e.g., parental autonomy support) (Grolnick & Ryan, 1989). The literature clearly shows that environments supporting one’s needs foster increased levels of well-being and greater levels of internalized motivation (Ryan & Solky, 1996).

#### 1.1.2. Pro-Environmental Attitudes and Behaviors and SDT

Self-determination theory significantly contributed to our understanding of environmentally responsible behaviors in youths. Research provides extensive evidence demonstrating how different types of motivation have a distinct impact on the maintenance and integration of PEB in different age groups. More specifically, a stronger self-determined motivation to protect the environment is associated with pro-environmental attitudes in children (Baierl & Bogner, 2023) and young adults (Juma-Michilena et al., 2023; Lavergne & Pelletier, 2015). Young adults with greater levels of self-determined motivation engage more frequently and persistently in PEB, even in conflicting or adverse environments (e.g., limited resources, more demanding PEB) (Baxter & Pelletier, 2020; Ryan & Deci, 2017). Self-determined motivation to protect the environment is also associated with more positive and negative effects on the environment (Pelletier et al., 1997), greater satisfaction toward the environment (Pelletier et al., 1996), greater knowledge about the environment (Juma-Michilena et al., 2023), and perceived competence for engaging in PEB (Pelletier et al., 1998; Tabernero & Hernández, 2011).

In addition to motivation, the satisfaction of basic psychological needs is also associated with PEB in young adults (Wray-Lake et al., 2017) and sufficiency-oriented consumption in the general population (Tröger et al., 2021). It was also found that adults who reported higher levels of needs satisfaction engaged in more PEB (i.e., purchasing local foods and buying environmentally-friendly gifts; Kasser & Sheldon, 2002). Other studies found that adolescents who have greater levels of happiness, as well as adults who reported higher levels of life satisfaction and positive affect, report engaging in more PEB (K. W. Brown & Kasser, 2005). Although the directionality of the association between needs satisfaction and environmental behaviors remains to be established (Kasser, 2009), these results provide evidence of the relevance of basic psychological needs in the environmental domain. Nonetheless, in this study, we propose to investigate whether the need for security, central to attachment theory, interacts with the basic needs identified by SDT in creating a favorable socioemotional context fostering PEBs in youths.

### 1.2. Attachment Theory

Attachment theory provides a compelling theoretical framework to understand youths’ emotions and behaviors in response to the climate crisis. Broadly speaking, attachment theory is interested in the development of affective relationships between humans and how these relationships help equip children and youth to deal with threats in their environment (Bowlby, 1969, 1982). Attachment theory identifies the need for security as a basic psychological need that drives children to seek proximity with significant caregivers as a secure base from which to explore their environment (Ainsworth, 1985; Bowlby, 1969, 1982; Grossmann et al., 2008; Sroufe & Fleeson, 1988). When children can rely on their caregivers in times of need or distress and feel safe to explore their environment, they develop a “secure attachment”. Through their relationship with the caregiver, securely attached children develop self-confidence and emotion regulation strategies that help them face perceived threats throughout their lives (Estevez et al., 2019; Karreman & Vingerhoets, 2012).

Some children, however, cannot rely on their caregivers for comfort and exploration based on the consistent rejection of a child’s bids for protection or the caregiver’s unavailability. Ainsworth et al. (1978) originally identified two insecure types: insecure-avoidant and insecure-resistant. Caregivers of avoidant children offer little comfort and protection when children are in distress. As a result, these children tend to minimize their distress to appear autonomous and avoid rejection by the attachment figure (Marvin et al., 2002). Caregivers of resistant children tend to be unpredictable in their response to their child (or lack thereof) and themselves show signs of anxiety. These children are more inclined to maximize their distress signals without deriving comfort from the caregiver’s reactions (Marvin et al., 2002). Attachment continues to be of relevance as children grow up, and consequently, a variety of measures, namely questionnaires, have been developed to study children’s attachment to their caregivers during adulthood. These questionnaires generally identify two main orientations corresponding to the original infant insecure patterns: Avoidance (minimization of attachment needs) and Anxiety (maximization of attachment needs) (Brennan et al., 1998; Fraley et al., 2000). Past research findings suggest that these insecure attachment patterns identified in emerging adults are associated with higher levels of distress (Wei et al., 2005).

#### 1.2.1. Pro-Environmental Attitudes and Behaviors and Attachment Theory

Given that attachment, at its core, deals with individuals’ reactions in the face of threats, it is possible that youths’ attachment relationships contribute to shaping whether they interpret climate change as a threat and whether they develop pro-environmental attitudes and behaviors. In one of the first studies examining attachment theory in the context of climate change, Nisa et al. (2021) showed that young adults are more likely to believe in climate change and to accept their personal responsibility towards it when they are primed to feel more securely attached to their caregivers. The study also showed that priming attachment increased empathy toward others and donations to environmental causes. Interestingly, they found that attachment-based messages were more effective in supporting pro-environmentalism than other types of messaging (e.g., reducing food waste). A second study found that climate-related stress was negatively associated with self-reported attachment security in German medical school students (Schwaab et al., 2022). A third study also showed that child-caregiver attachment security in Chinese school-aged children was associated with greater awareness of the risks posed by climate change (Zhong et al., 2021). While these early findings are promising and indicate that child-caregiver attachment may be an important factor to consider, this young literature is limited by an exclusive focus on attachment security, therefore overlooking the different types of insecurity, which may respond differently in the face of climate-related threats. On one hand, avoidant youth may minimize the importance of the climate crisis and downplay their ability to mitigate climate change. Their wariness of others may lead them to doubt our collective ability to make meaningful contributions to address climate change. On the other hand, youth with an anxious attachment may be extremely preoccupied with climate change. Although they might intend to help, their anxiety may hinder the adoption of PEB and attitudes.

#### 1.2.2. Need Satisfaction and Attachment Security

SDT and attachment theory share common qualities and are often viewed as complementary theories. First, both theories recognize the importance of socioemotional relations with significant others. For example, Grolnick (2003) suggested that children’s need for relatedness is fulfilled by a secure relationship with their caregivers. Both theories support the importance of the caregiver’s sensitivity in establishing healthy autonomy in children (Bowlby, 1973) and promote a person’s satisfaction with basic psychological needs. While secure attachment is defined as the balance between safety (relatedness) and exploration (autonomy), Deci and Ryan (2012) argue that the need for autonomy and relatedness are not inherently antagonistic but rather mutually supportive. This connection between autonomy support and intimacy is described as a lifelong dynamic (Ryan, 1993). According to both theories, a caregiver restricting child autonomy or rejecting the child’s need for relatedness likely fosters attachment insecurity and frustration.

Second, both theories share an interest in regulatory mechanisms. According to attachment theory (Sroufe et al., 1983), infants are not capable of regulating their own emotions and need the help of a caregiver to learn to manage their emotions. Conversely, SDT states that an individual’s behavioral regulation is based on caregivers supporting the child’s autonomy, facilitating their competence, and providing a structure (Deci & Ryan, 1985; Ryan & Deci, 2017). Therefore, despite differing emphases on safety and exploration, both theories underscore the crucial role of caregivers in shaping how children learn to regulate their emotions and behaviors, a notion particularly important in the study of youths’ responses to the threat of climate change.

Numerous studies have explored the associations between attachment orientations and need satisfaction in emerging adults. These studies found that greater satisfaction of the three psychological needs predicts greater security of attachment to multiple attachment figures (La Guardia et al., 2000). Other studies found associations between attachment as applied to specific significant relationships (e.g., parents, romantic partner, coach) and basic needs satisfaction (Felton & Jowett, 2013, 2017; Patrick et al., 2007; Wei et al., 2005).

### 1.3. Limitations of the Existing Research

Past research has focused on the association between attachment security and needs satisfaction as predictors of various psychological outcomes (La Guardia et al., 2000; Rockafellow, 2006; Wei et al., 2005). While some researchers include need satisfaction as a predictor of attachment (La Guardia et al., 2000), others include attachment as a predictor of need satisfaction (Felton & Jowett, 2017; López, 2022; Wei et al., 2005). By relying on a variable-centered approach, these studies provide important insight regarding the association between need satisfaction and attachment as well as a measure of the variance explained by each predictor. However, in doing so, they provide a measure of the association between the variables but fail to consider the heterogeneity existing within their sample. As such, we know very little about the various patterns that subgroups of participants may exhibit in their data. In a context where we have yet to understand why motivational interventions meant to increase the frequency of PEB fail for some individuals, relying on a person-centered approach can provide meaningful insight and help us identify and distinguish subgroups with specific patterns on targeted factors. As the association between need satisfaction and attachment security in previous studies is often modest at a sample level, it is likely that the strength of that association would vary in intensity within subgroups of participants and, in turn, impact their PEB. Beyond the limitations imposed by the use of a variable-centered approach, the role of attachment avoidance and anxiety has yet to be investigated in the environmental domain.

### 1.4. Current Study

The overarching goal of this study was to examine the joint impact of attachment (i.e., anxiety and avoidance) and need satisfaction on individuals’ motivational pathways in the environmental domain. For this goal, we relied on latent profile analysis, a person-centered statistical analysis designed to identify, within heterogeneous samples, latent clusters of individuals who display similarities on multiple variables of interest (Berlin et al., 2014; Nylund-Gibson & Choi, 2018). This approach was selected as it allows us to portray individual relational profiles instead of only measuring and comparing the impact of each variable and subsequently assessing their impact in the environmental domain. This is especially important considering that attachment and need satisfaction are two interrelated concepts taking root in child-caregiver relationships that influence how youths regulate their emotions and face adversity. Moreover, both theories reflect one’s perception of one’s social environment. In exploring both attachment and SDT theory, this study seeks to explain why youths with different relational profiles may show various feelings, motivations, and behaviors toward the environment. The questions investigated in this study are exploratory. First, no previous studies have explored the insecure subtypes (i.e., avoidant and anxious) in relation to need satisfaction and in an environmental context. Therefore, we do not know what specific profiles will emerge. Nonetheless, we can expect that profiles with a higher rate of security and/or need satisfaction will be associated with more positive feelings toward the environment, more self-determined motivation to protect it, and a higher frequency of pro-environmental behaviors. Second, no studies to date have assessed the different pathways leading to PEB according to the participants’ profiles. Therefore, this question remains exploratory.

## 2. Materials and Methods

### 2.1. Participants

The analytical sample comprised 1527 undergraduate students. On average, participants were aged 18.94 years (*SD* = 1.50, range = 17–26). Most participants identified as women (72.6%, *n* = 1108), 401 identified as men (26.3%), and 18 participants did not identify as any gender (1.2%). Most participants identify as White (58.7%), with the rest identifying as Black (12.1%), Asian (14.0%), Latina/Hispanic (1.5%), Middle Eastern (7.4%), Indigenous (0.8%), or another ethnicity (5.5%). Finally, 41.9% of participants reported living at home with their parents.

### 2.2. Procedure

Data collection took place from October to December 2023. Undergraduate students at an Eastern Canadian university were invited to participate in an online research participation pool. The system allowed students enrolled in introductory or second-year classes in psychology, communication, linguistics, or business administration from any program of study to participate. Participants provided informed consent before responding to a series of online questionnaires. Participants were granted 1% in course credit for participating in the survey, regardless of completion percentage. All procedures were approved by the university’s Research Ethics Board (H-08-23-9547) and met the ethical standards set out by the 1964 Declaration of Helsinki and its later amendments.

### 2.3. Measures

#### 2.3.1. Attachment Avoidance and Anxiety

Youth’s attachment orientations toward their mother and father were assessed separately using the Experiences in Close Relationships–Relationship Structures questionnaire (ECR-RS; Fraley et al., 2011). The ECR-RS is a validated nine-item measure of attachment avoidance and anxiety in different relationships (Fraley et al., 2011). Participants responded to the items using a Likert scale ranging from 1 (strongly disagree) to 7 (strongly agree). Attachment anxiety is assessed with three items assessing fear of rejection (e.g., “I’m afraid that this person may abandon me”). Attachment avoidance is assessed with six items addressing discomfort with closeness and dependency (e.g., “I do not feel comfortable opening up to this person”). The ECR-RS subscales show good internal consistency in all relational domains (Fraley et al., 2015) and excellent test–retest reliability for the parental domain (Fraley et al., 2011). In the current study, the internal consistency was excellent toward mothers (ω_t_ = 0.94) and fathers (ω_t_ = 0.95).

#### 2.3.2. Basic Need Satisfaction and Frustration

Basic psychological needs were assessed using the validated 24-item Basic Psychological Need Satisfaction and Frustration Scale (BPNSFS; B. Chen et al., 2015). Items were rated on a five-point Likert scale ranging from 1 (not true at all) to 5 (completely true). It is composed of three subscales that measure both the satisfaction and frustration of the need for autonomy (e.g., “*I feel a sense of choice and freedom in the things I undertake*” and “*I feel forced to do many things I would not choose to do*”), relatedness (“*I feel connected with people who care for me, and for whom I care*” and “*I feel the relationships I have are just superficial*”), and competence (“*I feel capable at what I do*” and “*I feel insecure about my abilities*”). Due to multicollinearity between the three subscales (*rs* = [0.73, 0.81]), a sole mean need satisfaction composite score was computed using all the scale items. Internal consistency was excellent for the need satisfaction composite score (ω_t_ = 0.92).

#### 2.3.3. Motivation Toward the Environment

Motivation underlying participants’ actions for the environment was assessed using a short form of the Motivation Towards the Environment Scale (MTES; Pelletier et al., 1998). The short form MTES is based on six items drawn from the original 24-item version of the MTES. The items cover intrinsic motivation, integrated regulation, identified regulation, introjected regulation, external regulation, and amotivation. Participants indicated their level of agreement for each item using a scale ranging from 1 (does not correspond at all) to 7 (corresponds exactly). Scores of intrinsic, integrated, and identified regulations were averaged to form a composite score representing self-determined motivation. The remaining regulation subtypes (introjected, external, and amotivation) items were averaged and transformed into a composite score representing non-self-determined motivation. In this study, the internal consistency of the scale was adequate (ω_t_ = 0.79).

#### 2.3.4. Environmental Emotions

Environmental emotions were assessed using the How Do You Feel About Environment questionnaire (Pelletier et al., 1996). Items were four positive adjectives (i.e., secure, serene, satisfied, and optimistic) and eight negative adjectives about one’s environmental emotions (i.e., discouraged, worried, pessimistic, helpless, frustrated, apprehensive, anxious, and ashamed). Participants were asked to rate their feelings on a seven-point Likert response scale from 1 (Not at all) to 7 (Extremely). For the present study, positive adjectives were grouped in a positive emotions composite score, and negative adjectives were grouped in a negative composite score (ω_t_ = 0.88).

#### 2.3.5. Environmental Attitudes

Participants’ environmental attitudes were assessed using the eight-item Pro-environmental Attitude Strength Scale (Lavergne & Pelletier, 2015). Two items measured participants’ views about the environment (e.g., “Human activities have a harmful impact on the environment”), and six items measured participants’ attitude strength (e.g., “How important to you personally are environmental issues”). Participants responded using a seven-point Likert scale from 1 (Not at all) to 7 (Very much). A composite score was obtained by averaging all environmental attitudes items, which had adequate internal consistency (ω_t_ = 0.77).

#### 2.3.6. Pro-Environmental Behaviors

The frequency of PEB in daily life was assessed using nine items of the Frequency of Recent Pro-Environmentally Relevant Actions Scale (Lavergne & Pelletier, 2015). Participants were asked to select the number of times they performed the listed actions (e.g., “*Purchased local foods*”) over the past week/7 days. Two items were dropped (i.e., “*Used a clothesline or drying rack*”; “*Opted to walk, cycle, or skate*, *instead of riding in a bus/car over a short distance (<3 km)*”) as they were weakly correlated (*r* < 0.30; T. A. Brown, 2006) with other items (*rs* = [0.04; 0.25]). The relative frequency of recent PEB was obtained by averaging the reported frequency for all environmental behaviors (ω_t_ = 0.79).

#### 2.3.7. Generalized Anxiety

Generalized anxiety symptoms were measured using the Generalized Anxiety Disorder Scale (GAD-7; Spitzer et al., 2006), a seven-item self-reported questionnaire based on the DSM-IV definition of generalized anxiety. Participants were asked to rate how often they were bothered by given symptoms during the last two weeks on a scale from 0 (not at all) to 3 (nearly every day). Items include statements such as “*feeling nervous, anxious or on edge*” and “*not being able to stop or control worrying*”. The total score ranged from 0 to 21, with higher scores indicating higher levels of generalized anxiety symptoms. The GAD-7 has excellent internal consistency, good test–retest reliability, and convergence and discriminant validity (Spitzer et al., 2006). For the current study, the measure showed excellent internal consistency (ω_t_ = 0.91).

### 2.4. Analytical Plan

Before conducting the analysis, the data were screened for insufficient effort responding, missing data, and data normality. Based on the work of Huang et al. (2012) and Curran (2016), the data were sequentially screened using multiple approaches to identify participants who may not have responded truthfully or attentively (i.e., Mahalanobis Distance, validation questions, and long string). Missing data were imputed using the *mice* package (van Buuren & Groothuis-Oudshoorn, 2011). The variables were thus transformed using a box–cox transformation using the *MASS* package (version 7.3-65; Venables & Ripley, 2002). After ensuring the normality of the distribution and applying the required transformation, the scores of the need satisfaction, attachment anxiety, and avoidance with the mother, as well as with the father, were standardized and winsorized to z = +/− 3.29. Once the profiles were generated, the same imputation process was applied to the outcome variables. The variables used to generate the latent profiles were not included in the second imputation process to avoid inflating the association between the outcomes and the profiles. For the full description of the data preparation procedure, refer to Appendix A. Descriptive statistics are summarized in Table 1.

Following preliminary analyses, we first performed a latent profile analysis to investigate underlying profiles in terms of basic psychological needs and attachment. To decrease the probability of a local solution, the model was generated using 7500 random starts, 1000 iterations by random start, and by retaining the 200 best solutions for the final optimization stage. The model was estimated using robust maximum likelihood (ML). We relied on a stepwise approach to determine the number of latent profiles. We thus started with a two-profile solution and iteratively added profiles while inspecting the fit indices at each step (Nylund et al., 2007; Spurk et al., 2020). The number of latent profiles was chosen to ensure a parsimonious solution, as well as to minimize the Bayesian information criterion (BIC), integrated complete-data likelihood criterion (ICL), log-likelihood, and the bootstrapped log-likelihood ratio, and maximize the entropy. However, it is worth noting that the BLRT is known to overestimate the number of profiles that need to be extracted (Morin & Marsh, 2015). To ensure the representativity of each profile, we ensured that the sample size of the smallest profile represented at least 3% of the sample (Spurk et al., 2020). The final decision also took into account theoretical considerations and sought to avoid profile redundancy.

Second, in order to investigate whether the identified profiles were associated with meaningful differences in levels of environmental self-determined motivation, non-self-determined motivation, environmental emotions, environmental attitudes, and frequency of environmental behaviors, a MANOVA was conducted, controlling for the effect of generalized anxiety. Although the MANOVA can highlight between-profile differences in terms of observed scores on each outcome, it does not provide information on differences between latent profiles in terms of underlying motives for engaging in environmental behaviors. Consequently, to investigate between-profile differences in terms of their underlying motivational pathway (i.e., the motives underlying the frequency of their environmental behaviors), we performed a multigroup path analysis. The model was estimated using full-information ML with 5000 bootstrapped samples to obtain a 95% confidence interval. The goodness of fit of the path model was assessed with multiple fit indices (robust CFI ≥ 0.95; SRMR ≤ 0.08; robust RMESA ≤ 0.05) (Hu & Bentler, 1999). The data were deemed to provide an adequate fit to the multigroup path model if constraining the model led to an increase in RMSEA ≤ 0.015 and CFI ≤ 0.01 (F. F. Chen, 2007). The model fit was first assessed at an omnibus level before conducting the multigroup analysis. All analyses were conducted on *R* (version 4.4.1; R Core Team, 2024) using the psych (Revelle, 2024), tidyLPA (Rosenberg et al., 2018), and lavaan (Rosseel, 2012) packages.

## 3. Results

### 3.1. Latent Profile Analysis

#### 3.1.1. Procedure

The latent profile analysis was then conducted using five variables (i.e., avoidance with the (1) mother and (2) father, anxiety with the (3) mother and (4) father, and (5) needs satisfaction). A series of models were compared in order to select the model offering the best model fit. The model solutions ranged between two and nine profiles. Fit indices (i.e., BIC, ICL, AIC, SABIC) indicated that a seven-profile solution offered the best fit to the data, as displayed in Table 2. The BLRT could not be computed for the four-, eight- and nine-profile solutions, suggesting possible convergence issues for these solutions. All solutions offered an adequate entropy (>80).

#### 3.1.2. Identification of the Number of Profiles

When generating a two-profile solution, two distinct profiles emerged (insecure/low-need satisfaction vs secure/high-need satisfaction). Adding a third or fourth profile decreased the BIC, SABIC, LL, and AIC. The three-profile solution did not propose a qualitatively distinct profile, as the model solely yielded an additional quantitatively distinct profile. The third profile was solely distinguishable from the insecure/low-need satisfaction profile in terms of the observed intensity on each predictor (lower level of insecurity and similar level of basic psychological need satisfaction). A four-profile solution was needed to obtain an additional qualitatively and quantitatively distinct profile (i.e., higher attachment avoidance, lower attachment anxiety). Five-profile and six-profile solutions were also investigated, but increasing the number of profiles beyond four did not result in additional qualitatively distinct profiles. No further solutions were assessed as the sample size of the smallest profile was below 3%; despite issues surrounding the BLRT, we selected a four-profile solution as the final model.

#### 3.1.3. Interpretation of Extracted Profiles

**Figure 1:**
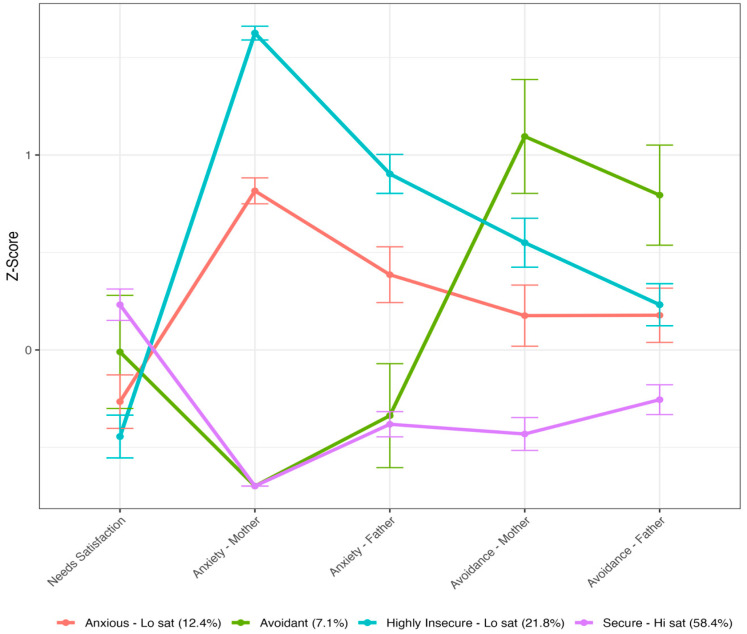
Results from the Latent Profile Analysis. Error bars represent the 95% confidence interval.

As represented in Figure 1, the first profile extracted (*n* = 109; 7.1%) included participants with lower levels of attachment anxiety and higher levels of avoidance of both caregivers. Participants in this profile displayed somewhat moderate levels and more variance around the estimate of need satisfaction. This profile can be characterized as our *avoidant* group. The second profile extracted (*n* = 892; 58.4%) was characterized by low levels of anxiety and avoidance with both caregivers, as well as higher need satisfaction. This profile can be characterized as our secure/high-need satisfaction group. The third profile extracted (n = 190; 12.4%) showed moderately high levels of attachment anxiety but close to average levels of avoidance with both caregivers. Participants in this profile also had lower levels of need satisfaction. This profile was characterized as our anxious/low-need satisfaction group. The fourth profile extracted (n = 333; 21.8%) showed high levels of anxiety and avoidance for both caregivers. Participants from this profile also had lower levels of satisfaction with needs. This profile was characterized as our highly insecure/low-need satisfaction group.

### 3.2. Comparison of Extracted Profiles

After generating the profiles, a MANOVA was performed to test between profile differences in terms of the level of self-determined and non-determined motivation, environmental attitudes’ strength, positive and negative emotions, and PEB frequency while controlling for the effect of generalized anxiety. Omnibus results suggest that participants’ latent profiles had a significant effect on the outcomes (*F*_(21, 4548)_ = 6.38, *p* < 0.001, η^2^ = 0.03). After accounting for the difference in generalized anxiety (*F*_(3, 1520)_ = 30.08, *p* < 0.001, η^2^ = 0.056) across profiles, the analysis revealed a significant effect of the profiles on participants’ levels of self-determined motivation (*F*_(3,1520)_ = 3.73, *p* = 0.011, η^2^ = 0.007), non-self-determined motivation (*F*_(3,1520)_ = 5.72, *p* < 0.001, η^2^ = 0.011), positive emotions (*F*_(3,1520)_ = 4.41, *p* = 0.004, η^2^ = 0.009), negative emotions (*F*_(3,1520)_ = 9.40, *p* < 0.001, η^2^ = 0.022) towards the environment, and PEB frequency (*F*_(3,1520)_ = 2.62, *p* = 0.049, η^2^ = 0.005). There was, however, no statistically significant effect of participants’ latent profile for environmental attitudes strength (*F*_(3,1520)_ = 1.54, *p* = 0.202, η^2^ = 0.003).

#### Post-Hoc Analyses

Pairwise comparisons were performed while controlling for the effect of generalized anxiety to identify significant between-profile differences amongst significant main effects. A Bonferroni correction was applied to decrease the type I error rate. Significant comparisons are presented below, but full results are presented in Table 3. Post-hoc comparisons revealed that participants from the secure/high-need satisfaction profile (*EMM* = 4.90, *SE* = 0.04) had significantly greater levels of self-determined motivation than participants from the highly insecure/low-need satisfaction (*EMM* = 4.70, *SE* = 0.06) and anxious/low-need satisfaction profiles (*EMM* = 4.62, *SE* = 0.08). Pairwise comparisons also revealed a significant difference in the reported frequency of PEB; participants from the anxious/low-need satisfaction (*EMM* = 3.31, *SE* = 0.12) profile reported engaging less frequently in PEB than the highly insecure/low-need satisfaction (*EMM* = 3.72, *SE* = 0.10) profile.

### 3.3. Path Analysis

**Figure 2:**
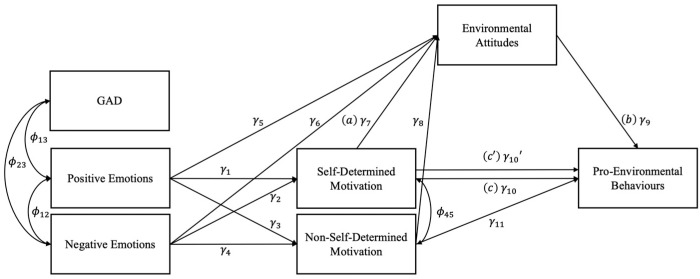
Hypothesized Mediated Path Model.

Before proceeding with the multigroup path analysis, the adequacy of the mediated path model was assessed at a sample level. Fit indices from the sample-level path analysis suggest that the model was a good fit to the data (χ^2^_(7)_ = 19.64, *p* = 0.006; robust CFI = 0.994; robust RMSEA = 0.034, CI_90_ = [0.017, 0.053]; SRMR = 0.017; results are summarized in Table 4). After constraining the profiles to the model, the model fit remained adequate (χ^2^_(28)_ = 35.74, *p* = 0.149, robust CFI = 0.996, robust RMSEA = 0.027, CI_90_ = [0.000, 0.051], SRMR = 0.024; see Figure 2).

#### 3.3.1. Effects on Self-Determined Motivation Toward the Environment

Secure/high-need satisfaction profile. Self-determined motivation was positively predicted by negative (β = 0.45, *p* < 0.001, CI_95_ [0.37, 0.53]) and positive emotions (β = 0.22, *p* < 0.001, CI_95_ [0.14, 0.30]) in the secure/high-need satisfaction profile. The model explained 12.7% of the variance in self-determined environmental motivation.

Avoidant profile. As it regards the avoidant profile, self-determined motivation was predicted by negative emotions (β = 0.41, *p* = 0.001, CI_95_ [0.17, 0.65]) but was not predicted by positive emotions (β = 0.23, *p* = 0.065, CI_95_ [−0.01, 0.46]). The model explained 10.8% of the variance in self-determined environmental motivation.

Anxious/low-need satisfaction profile. A similar pattern was observed in the anxious/low-need satisfaction profile. Again, self-determined motivation was mainly predicted by negative emotions (β = 0.32, *p* < 0.001, CI_95_ [0.16, 0.48]) and, to a lesser extent, by positive emotions (β = 0.19, *p* = 0.037, CI_95_ [0.01, 0.36]). The model explained 8.0% of the variance in self-determined environmental motivation.

Highly insecure/low-need satisfaction profile. Similarly, self-determined motivation was mainly predicted by negative emotions (β = 0.30, *p* < 0.001, CI_95_ [0.18, 0.42]) and, to a lesser extent, by positive emotions (β = 0.13, *p* = 0.038, CI_95_ [0.01, 0.26]) in the highly insecure/low-need satisfaction profile. The model explained 6.5% of the variance in self-determined environmental motivation for this profile.

#### 3.3.2. Effects on Non-Self-Determined Motivation Towards the Environment

Secure/high-need satisfaction profile. The non-self-determined motivation was positively predicted by negative emotions (β = 0.28, *p* < 0.001, CI_95_ [0.20, 0.36]) and by positive emotions (β = 0.20, *p* < 0.001, CI_95_ [0.12, 0.28]) in the secure/high-need satisfaction profile. The model explained 4.9% of the variance in non-self-determined environmental motivation.

Avoidant profile. As it pertains to the avoidant profile, non-self-determined motivation was predicted by negative emotions (β = 0.37, *p* = 0.002, CI_95_ [0.16, 0.59]) and positive emotions (β = 31, *p* = 0.003, CI_95_ [0.11, 0.51]). The model explained 9.3% of the variance in non-self-determined environmental motivation.

Anxious/low-need satisfaction profile. Contrary to what has been observed in the other profiles, positive (β = 0.17, *p* = 0.063, CI_95_ [−0.01, 0.36]) and negative (*β* = 0.11, *p* = 0.248, CI_95_ [−0.08, 0.29]) emotions did not significantly predict non-self-determined motivation in the anxious/low-need satisfaction profile. The model explained 2.3% of the variance in non-self-determined environmental motivation.

Highly insecure/low-need satisfaction profile. Non-self-determined motivation was predicted by negative emotions (β = 0.37, *p* < 0.001, CI_95_ [0.24, 0.49]) and positive emotions (β = 0.34, *p* < 0.001, CI_95_ [0.22, 0.45]). The model explained 13.0% of the variance in non-self-determined environmental motivation.

#### 3.3.3. Effects on Environmental Attitudes

Secure/high-need satisfaction profile. Environmental self-determined motivation (*β* = 0.50, *p* < 0.001, CI_95_ [0.45, 0.55]) and negative emotions (β = 0.29, *p* < 0.001, CI_95_ [0.22, 0.36]) significantly positively predicted environmental attitudes. Non-self-determined motivation (β = −0.03, *p* = 0.372, CI_95_ [−0.08, 0.03]) and positive emotions (β = 0.00, *p* = 0.928, CI_95_ [−0.06, 0.07]) were not associated with environmental attitudes in the secure/high-need satisfaction profile. The model explained 41.7% of the variance in environmental attitudes.

Avoidant profile. Environmental self-determined motivation (*β* = 0.37, *p* < 0.001, CI_95_ [0.21, 0.52]) and negative emotions (*β* = 0.47, *p* < 0.001, CI_95_ [0.27, 0.66]) significantly positively predicted environmental attitudes, while non-self-determined motivation (*β* = −0.14, *p* = 0.020, CI_95_ [−0.26, −0.02]) significantly negatively predicted environmental attitudes. Positive emotions (β = 0.11, *p* = 0.267, CI_95_ [−0.09, 0.31]) were not associated with environmental attitudes in the avoidant profile. The model explained 36.1% of the variance in environmental attitudes.

Anxious/low-need satisfaction profile. In the anxious/low-need satisfaction profile, environmental self-determined motivation (β = 0.64, *p* < 0.001, CI_95_ [0.57, 0.72], non-self-determined motivation (β = 0.09, *p* = 0.041, CI_95_ [0.00, 0.18]), and negative emotions (β = 0.24, *p* < 0.001, CI_95_ [0.13, 0.35]) predicted environmental attitudes. The analysis did not reveal a significant effect of positive emotions (β = −0.07, *p* = 0.233, CI_95_ [−0.19, 0.05]) on environmental attitudes. The model explained 57.1% of the variance in environmental attitudes.

Highly insecure/low-need satisfaction profile. Environmental self-determined motivation (β = 0.57, *p* < 0.001, CI_95_ [0.51, 0.63]) and negative emotions (β = 0.24, *p* < 0.001, CI_95_ [0.15, 0.33]) significantly positively predicted environmental attitudes. However, results suggest that environmental attitudes are not predicted by environmental non-self-determined motivation (β = −0.01, *p* = 0.765, CI_95_ [−0.10, 0.07]) and positive emotions (β = −0.08, *p* = 0.080, CI_95_ [−0.18, 0.01]) in the highly insecure/low-need satisfaction profile. The model explained 48.2% of the variance in environmental attitudes.

#### 3.3.4. Effects on Frequency of Environmental Behaviors

Secure/high-need satisfaction profile. The frequency of PEB was predicted by the direct effects of environmental self-determined motivation (β = 0.07, *p* = 0.048, CI_95_ [0.01, 0.14]) and environmental attitudes (β = 0.15, *p* < 0.001, CI_95_ [0.08, 0.22]) in the secure/high-need satisfaction profile. The analysis did not reveal a significant direct effect of non-self-determined motivation (β = −0.02, *p* = 0.449, CI_95_ [−0.09, 0.04]) on environmental behaviors. The analysis revealed a partial mediation as demonstrated by a significant indirect effect of self-determined motivation through environmental attitudes (β = 0.08, *p* < 0.001; CI_95_ [0.04, 0.11]) and a significant total effect (β = 0.15, *p* < 0.001; CI_95_ [0.08, 0.21]). Results suggest that self-determined motivation is associated with the frequency of PEB beyond its association with environmental attitudes. The model explained 4.1% of the variance in environmental behaviors.

Avoidant profile. The analysis did not reveal any significant direct effect on environmental behaviors in the avoidant profile. The direct effects of environmental self-determined motivation (β = −0.04, *p* = 0.703, CI_95_ [−0.18, 0.26]), non-self-determined motivation (β = 0.10, *p* = 0.127; CI_95_ [−0.03, 0.24]), and environmental attitudes (β = 0.12, *p* = 0.200, CI_95_ [−0.06, 0.30]) were non-significant. The indirect effect of self-determined motivation through environmental attitudes (β = 0.04, *p* = 0.241; CI_95_ [−0.02, 0.11]) and the total effect of self-determined motivation was non-significant (β = 0.09, *p* = 0.162; CI_95_ [−0.11, 0.28]). The model explained 3.2% of the variance in environmental behaviors.

Anxious/low-need satisfaction profile. The analysis did not reveal any significant direct effect on environmental behaviors in the anxious/low-need satisfaction profile. The direct effects of self-determined motivation (β = −0.02, *p* = 0.862, CI_95_ [−0.23, 0.19]) and non-self-determined motivation (β = −0.12, *p* = 0.077, CI_95_ [−0.24, 0.01]) on environmental behaviors were non-significant. In contrast, the direct effect of environmental attitudes (β = 0.21, *p* = 0.036, CI_95_ [0.01, 0.40]) on environmental behaviors was significant. The indirect effect of self-determined motivation through environmental attitudes (*β* = 0.13, *p* = 0.038; CI_95_ [0.01, 0.26]) was statistically significant, whereas the total effect of self-determined motivation (β = 0.11, *p* = 0.135, CI_95_ [−0.04, 0.26]) was not statistically significant. The model explained 4.6% of the variance in environmental behaviors.

Highly insecure/low-need satisfaction profile. There was a significant direct effect of environmental attitudes (β = 0.20, *p* < 0.001, CI_95_ [0.09, 0.31) on the frequency of environmental behaviors. The analysis did not reveal a significant direct effect of self-determined (β = −0.04, *p* = 0.530, CI_95_ [−0.16, 0.08]) and non-self-determined motivation (β = −0.06, *p* = 0.297, CI_95_ [−0.18, 0.05]) on environmental behaviors. There was a statistically significant indirect effect of self-determination through environmental attitudes (β = 0.11, *p* = 0.001, CI_95_ [0.05, 0.18]). The total effect of environmental self-determined motivation (β = 0.07, *p* = 0.176, CI_95_ [−0.03, 0.18]) on the frequency of environmental behaviors was non-significant. The model explained 3.3% of the variance in environmental behaviors.

## 4. Discussion

While extensive research has examined why people choose to engage in PEB, the literature on environmental motivation research has yet to fully understand why some people do not engage in PEB despite being motivated or having strong environmental attitudes (Carrington et al., 2014; Farjam et al., 2019; Lavergne & Pelletier, 2015). In this project, we proposed that attachment security and need satisfaction may play a pivotal role in shaping pro-environmental attitudes and behaviors. We investigated (a) the association between need satisfaction and attachment using a person-centered approach, (b) between-profile differences in terms of environmental variables (e.g., motivation, attitudes, behaviors), and (c) whether latent profiles were associated with different motivational pathways. Results shed light on the impact of attachment orientations on the association between environmental motivation and PEB beyond the role of need satisfaction. While the association between motivation and PEB is deemed to be universal in the motivation literature, results from the present study depict a much more complex association between these variables.

### 4.1. Latent Profiles of Need Satisfaction and Attachment

Our latent profile analysis revealed four profiles characterized by varying levels of attachment insecurity and satisfaction of basic psychological needs. The profiles are coherent with theoretical expectations. First, the three attachment styles (i.e., security, avoidance, and anxiety) are distinguished in separate profiles, with an additional profile combining high rates of both types of insecurity. This profile could be indicative of a disorganized attachment characterized by a combination of two conflicting tendencies (i.e., minimization and maximization of attachment signals; Main & Solomon, 1990). In addition, we found that higher levels of attachment security were associated with higher satisfaction of basic psychological needs, whereas higher levels of anxiety and avoidance were associated with lower satisfaction of basic psychological needs. These findings are consistent with previous studies examining the association between attachment and need satisfaction (Felton & Jowett, 2013; Wei et al., 2005). In addition, our results also show variability in the intensity of the association between attachment and need satisfaction in the profiles. For example, the avoidant profile has a greater variability of need satisfaction compared to the other groups. The fact that some participants in the avoidant profile reported high-need satisfaction is coherent with their tendency to present a glorified version of their relationship and minimize their impact on their own functioning (Hesse, 2008).

#### 4.1.1. Between-Profile Differences

Although the latent profile analysis reliably produced four distinct profiles, our results counterintuitively revealed that they had similar characteristics in many aspects. For instance, there were no differences among profiles in terms of positive and negative emotions about the environment. Notable differences emerged only for a few variables, specifically when comparing environmental behaviors as well as when comparing levels of self-determined motivation. However, while this absence of differences indicates somewhat homogeneous mean distributions across most variables, the subsequent path analyses revealed important nuances when examining the associations between these variables. Therefore, while groups might appear similar at the between-profile level, the important distinctions lie in their different motivational pathways.

#### 4.1.2. Motivational Pathways

Results from the multigroup path analysis shed light on the different ways variables interact across latent profiles, suggesting that individual differences in terms of attachment security and need satisfaction may be associated with distinct paths leading to these outcomes. For instance, self-determined motivation was a direct predictor of PEB solely in the secure high-need satisfaction profile. The association between self-determined motivation and PEB is in line with previous studies (Baxter & Pelletier, 2020; Juma-Michilena et al., 2023; Lavergne & Pelletier, 2015; Ryan & Deci, 2017). However, it adds an important nuance by suggesting this association may be stronger in the context of attachment security and need satisfaction. According to both theories, supportive parenting offers a context that fosters both attachment security and needs satisfaction (Whipple et al., 2009), which may help youth develop an internal locus of causality. Altogether, this may nurture a sense of coherence in individuals, explaining the stronger association between their self-determined motivation and their PEB.

Another important finding is the indirect effect of self-determined motivation on the frequency of PEB operating through environmental attitudes. For all profiles, except for the avoidant one, self-determined motivation was a significant and positive antecedent of PEB mediated through environmental attitudes. In the case of the secure profile, this indirect pathway was present in addition to the direct pathway discussed above, therefore predicting PEB in two separate ways. For the anxious/low-need satisfaction and highly insecure/low-need satisfaction profiles, only the indirect pathway was significant, showing a predictive effect from self-determined motivation to PEB through attitudes. This second pathway, significant across three different profiles, highlights the key role played by environmental attitudes in predicting PEB. Finally, in the case of the avoidant profile, no significant pathway was identified, which is consistent with the traditional view that avoidantly attached individuals present behaviors and attitudes that aim to minimize threats or importance of relationships and, as a result, may display apparently contradictory behaviors (Marvin et al., 2002). For example, they may seek someone’s company while pretending they do not really care for that person, or, in the case of the current study, they may display PEB but claim they do not believe in a climate crisis. Taken together, these findings suggest that environmental attitudes (e.g., how personally important environmental issues are) play a more salient role in individuals with lower attachment security. In other words, for participants in anxious/low-need satisfaction or highly insecure/low-need satisfaction profiles, self-determined motivation has an influence on PEB *through* environmental attitudes. While this process is also observed in participants from the secure/high-need satisfaction profile, the direct effect of self-determined motivation on PEB was also significant in this group.

In all profiles, with the exception of the avoidant ones, we found that participants exhibiting more negative and positive emotions toward the environment reported more self-determined motivation toward the environment. In the avoidant profile, negative emotions, but not positive emotions, were related to self-determined motivation. Findings were similar for non-self-determined motivation: in all profiles except for the anxious/low-need satisfaction one, we found that participants exhibiting more negative and positive emotions toward the environment reported more non-self-determined motivation toward the environment. These results are in line with Pelletier et al. (1997), who found that different negative emotions and positive emotions toward the environment were distinctly positively correlated with self-determined types of motivation toward the environment.

Overall, our findings have theoretical implications for the fields of attachment and SDT. First, the secure/high-need satisfaction profile was the only group to exhibit a coherent pattern across environmental attitudes, motivation to protect the environment, and pro-environmental behaviors. These results suggest that youth who experience healthy and satisfying relationships with their parents may develop a stronger sense of relatedness, autonomy, and competence—key psychological resources that support the translation of motivation into action. Second, our results suggest that, for youth with higher levels of attachment anxiety and low-need satisfaction, the perceived importance of environmental issues plays a crucial role in fostering higher levels of PEB. This is perhaps unsurprising, as anxious youth may be more reactive when they view the climate crisis as an imminent personal threat. However, when they distance themselves from environmental concerns—reflected in lower environmental attitudes—their motivation alone does not seem sufficient to drive PEB. Lastly, although youth in both the secure and avoidant profiles displayed similar levels of self-determined motivation and environmental attitudes, these factors do not seem to impact behavior among avoidant individuals. Our model did not fully capture the process underlying the adoption of PEBs in avoidant youths. While more research is required to understand this underlying process, from a theoretical standpoint, we can hypothesize that since these youths tend to minimize the importance of relationships, they may be more likely to perform PEBs for their own benefit (e.g., financial incentives, convenience); motives associated with non-self-determined motivation. Although the direct effect of non-self-determined motivation (NSDM) was not statistically significant in this group (*p* = 0.127), the relatively small sample size of the avoidant/low-need satisfaction profile (*n* = 109) may have limited our statistical power to detect meaningful effects.

#### 4.1.3. Implications for Environmental and Motivation Research

The results suggested that the direct effect of self-determined motivation on PEB was only significant in the secure profile, which comprised 58.4% of participants. The indirect effect of self-determination through environmental attitudes was significant in the secure, anxious, low-need satisfaction, and highly insecure low-need satisfaction profiles but not in the avoidant profile.

These results have implications for the field of environmental motivation, as the results suggest that the association between environmental motivation and subsequent PEB is modulated by attachment orientations. As it is estimated that around 40% of the adult population (Mickelson et al., 1997) has an insecure attachment style, these results may suggest that interventions targeting environmental self-determined motivation would be ineffective for a considerable proportion of the population. Interestingly, in spite of the differential motivational pathways, we did not identify significant differences across groups for the frequency of environmental behaviors, supporting the idea that there are multiple pathways leading to such behaviors. The present study highlights the relevance of considering inter-individual differences in motivational pathways in order to tailor environmental interventions in an effective manner. Participants in the secure/high-need satisfaction and the highly insecure/low-need satisfaction profiles were the only ones with significantly different levels of self-determined motivation (secure/high-need satisfaction > highly insecure/low-need satisfaction). These two profiles were also the only ones in which we found a significant indirect effect of self-determined motivation through environmental attitudes. Indeed, considering attachment allowed us to uncover heterogeneities in the initiation of PEB. The results suggest that solely securely attached and highly need-satisfied individuals derive their PEB directly from self-determined motivation for the environment.

Although more research is needed to understand how attachment contributes to the association between motivation and behaviors in the environmental domain, the results support that individuals with a secure attachment and high-need satisfaction might have more psychological and emotional resources to help them cope with the threat that the climate crisis represents. As a result, securely attached individuals might be less prone to feeling helpless and may feel more competent in the face of the climate threat. Lavergne and Pelletier (2015) demonstrated that in the face of incongruence between attitudes and behaviors in the environmental domain, individuals cope with their discomfort through behavioral change or cognitive restructuring. On a daily basis, cognitive restructuring is adaptive for individuals with insecure attachment styles, as it is associated with increased resilience (Terzi, 2013). Securely attached individuals may have the coping strategies required to translate their dissonance into actions rather than into disengagement.

The present results have implications for environmental researchers and policymakers. The findings suggest that interventions framing their message in ways that aim to increase PEB by fostering higher levels of self-determined motivation may not be effective for insecurely-attached/low-need satisfaction individuals. Results shed light on the restrictive nature of solely considering motivation when exploring the factors promoting environmental behaviors and the relevance of considering attachment in the environmental domain. Interestingly, these results might imply that individuals displaying attachment insecurity and low-need satisfaction are not used to acquiring their needs responded to by their environment and, therefore, are more inclined to rely on their own personal beliefs to drive their behaviors. Beliefs pertaining to the environmental domain, indeed, were the main predictor of PEB in these profiles, which could suggest that messages emphasizing the urgency of the climate fight may have a greater impact on their beliefs and PEBs, even if they are not showing a self-determined motivation to protect the environment.

#### 4.1.4. Limitations and Future Research

This study has limitations. The sample was composed of undergraduate students and may not be representative of the general population. The present research does not inform us that profiles based on need satisfaction and attachment insecurity might impact the motivational pathway for PEB in other populations. Future research would benefit from investigating the role of attachment in other populations’ environmental domains. Additionally, given the observational design of the study, it is impossible to determine whether interventions targeting self-determined motivation would lead to an increased frequency of PEB in participants with a secure/high-need satisfaction latent profile. Future research would benefit from relying on a longitudinal and/or experimental design to investigate whether latent profiles differ in terms of frequency of PEB across time and following a motivational intervention. It is also worth noting that the group size of certain profiles (i.e., *anxious/low-need satisfaction* and *avoidant*) was below the recommended size (*n* > 400) required to obtain a stable solution when conducting multigroup path analysis (Luong & Flake, 2023). It is possible that some effects from the multigroup path analysis may be unreliable due to a lack of statistical power. More research with larger samples is needed to investigate the stability of the present findings. Although the present research investigates differences across latent profiles as it pertains to PEB, it does not investigate how the profiles may differ in terms of motivational pathways for harmful environmental behaviors. As suggested by Desmarais (2019), pro- and harmful-environmental behaviors are two conceptually distinct concepts with distinctive motivational pathways. As a result, it is likely that profiles differ in terms of the association between motivation and harmful environmental behaviors. Additionally, as mentioned in the Methods Section, some items measuring PEB had low correlations with other items. Future research would benefit from replicating the present result using another measure of PEB. Globally, future research would benefit from relying on more encompassing and person-centered models to understand how the factors driving people to engage in environmental behaviors differ across individuals.

## 5. Conclusions

The present results offer insight into the prominent role of attachment in the environmental domain and promote the proximal association between need satisfaction and attachment theory. The findings support the relevance of considering attachment orientations when designing interventions promoting environmental behaviors. This study positions attachment theory and self-determination theory as useful frameworks to investigate the attitude–behavior gap in the environmental domain and as tools to understand why individuals differ in their responses to environmental threats.

## Figures and Tables

**Table 1:** Descriptive Statistics.

Variables	*M* (*SD*)	1	2	3	4	5	6	7	8	9	10	11
1—Anxiety—Mother	1.55 (1.12)	-										
2—Avoidance—Mother	3.30 (0.47)	0.33 *	-									
3—Anxiety—Father	1.79 (1.42)	0.55 *	0.19 *	-								
4—Avoidance—Father	3.42 (0.50)	0.15 *	0.60 *	0.35 *	-							
5—Need Satisfaction	3.61 (0.46)	−0.29 *	0.07 *	−0.27 *	0.06 *	-						
6—SDM	4.80 (1.15)	−0.05	0.04	0.00	0.03	0.23 *	-					
7—NSDM	3.30 (0.89)	0.09 *	0.15 *	0.13 *	0.12 *	−0.07 *	0.08 *	-				
8—Positive Emotions	3.16 (1.14)	−0.09 *	−0.03	−0.08 *	−0.04	0.23 *	−0.03	0.06 *	-			
9—Negative Emotions	4.44 (1.19)	0.14 *	0.13 *	0.15 *	0.12 *	−0.15 *	0.28 *	0.28 *	−0.58 *	-		
10—Attitudes	4.84 (0.82)	−0.01	0.06 *	0.02	0.06 *	0.11 *	0.60 *	0.07 *	−0.20 *	0.43 *	-	
11—Behaviors	3.59 (1.72)	0.02	0.04	0.01	−0.01	0.05 *	0.16 *	−0.02	−0.02	0.09 *	0.22 *	-
12—GAD	9.43 (5.86)	0.22 *	0.19 *	0.24 *	0.17 *	−0.31 *	0.11 *	0.15 *	−0.25 *	0.37 *	0.15 *	0.02

Notes. *n* = 1527. *SDM* = Self-determined motivation; *NSDM* = Non-self-determined motivation. *GAD* = Generalized Anxiety. * *p* < 0.05.

**Table 2:** Latent Profile Analysis Fit Indices.

n Profiles	LL	AIC	BIC	SABIC	ICL	Entropy	% Smallest Profile	BLRT	BLRT (*p*)
2	−9735.83	19,523.65	19,662.26	19,579.66	−19,726.51	0.92	0.11	303.26	0.00
3	−7853.22	15,770.44	15,941.03	15,839.38	−15,951.30	0.99	0.12	1815.86	0.00
4	−7832.84	15,741.69	15,944.27	15,823.55	−16,168.86	0.89	0.07	NC *	NC *
5	−6738.92	13,565.84	13,800.41	13,660.63	−13,833.51	0.98	0.08	330.38	0.00
6	−6716.92	13,533.83	13,800.38	13,641.55	−14,013.55	0.91	0.06	NC *	NC *
7	−6702.52	13,517.03	13,815.57	13,637.67	−14,054.89	0.91	0.01	49.43	0.00
8	−7479.37	15,082.73	15,413.26	15,216.30	−15,673.58	0.90	0.00	NC	NC
9	−6964.86	14,065.71	14,428.23	14,212.21	−14,699.72	0.90	0.00	NC	NC

Notes. * NC = Not computed.

**Table 3:** Pairwise Comparisons Across Latent Profiles.

		**Self-Determined Motivation**				**Non-Self-Determined Motivation**			
		*EMM* Δ	*SE*	*t*	*p*	*EMM* Δ	*SE*	*t*	*p*
Sec/HS	Avoidant	0.30	0.12	2.62	0.054	−0.12	0.09	−1.33	1.00
	Anx/LS	0.28	0.09	3.02	0.015 *	−0.18	0.07	−2.49	0.078
I confirm	High insec/LS	0.20	0.08	2.70	0.042 *	−0.13	0.06	−2.17	0.178
Avoidant	Anx/LS	−0.03	0.14	−0.20	1.00	−0.06	0.11	−0.53	1.00
	High insec/LS	−0.10	0.13	−0.79	1.00	−0.01	0.10	−0.08	1.00
Anx/LS	High insec/LS	−0.07	0.10	−0.69	1.00	0.05	0.08	0.61	1.00
		**Positive Emotions**				**Negative Emotions**			
		*EMM* Δ	*SE*	*t*	*p*	*EMM* Δ	*SE*	*t*	*p*
Sec/HS	Avoidant	−0.00	0.11	−0.32	1.00	0.04	0.11	0.37	1.00
	Anx/LS	0.07	0.09	0.82	1.00	−0.05	0.09	−0.53	1.00
	High insec/LS	0.10	0.07	1.35	1.00	−0.19	0.12	−2.58	0.061
Avoidant	Anx/LS	0.08	0.13	0.57	1.00	−0.09	0.13	−0.66	1.00
	High insec/LS	0.10	0.12	0.83	1.00	−0.23	0.12	−1.87	0.367
Anx./LS	High insec/LS	0.03	0.10	0.26	1.00	−0.14	0.10	−1.40	0.978
		**Environmental Behaviors**							
		*EMM* Δ	*SE*	*t*	*p*				
Sec/HS	Avoidant	−0.09	0.18	−0.53	1.00				
	Anx/LS	0.27	0.14	2.00	0.272				
	High insec/LS	−0.14	0.11	−1.25	1.00				
Avoidant	Anx/LS	0.37	0.21	1.78	0.447				
	High insec/LS	−0.05	0.19	−0.26	0.447				
Anx./LS	High insec/LS	−0.42	0.16	−2.66	0.048				

Notes. Sec/HS = Secure, high-need satisfaction profile; Anx/LS = Anxious, low-need satisfaction profile; High insec/LS = Highly insecure, low-need satisfaction profile.

**Table 4:** Results from the Omnibus Path Analysis.

	β	*SE*	*p*	95% CI
*Path Coefficients*				
SDM ← Positive Emotions (γ_1_)	0.20	0.03	<0.001	[0.14; 0.26]
SDM ← Negative Emotions (γ_2_)	0.39	0.03	<0.001	[0.33; 0.45]
NSDM ← Positive Emotions (γ_3_)	0.24	0.02	<0.001	[0.18; 0.30]
NSDM ← Negative Emotions (γ_4_)	0.30	0.02	<0.001	[0.24; 0.36]
Attitudes ← Positive Emotions (γ_5_)	−0.02	0.02	0.381	[−0.07; 0.02]
Attitudes ← Negative Emotions (γ_6_)	0.28	0.03	<0.001	[0.23; 0.33]
Attitudes ← SDM (γ_7_)	0.52	0.02	<0.001	[0.49; 0.56]
Attitudes ← NSDM (γ_8_)	−0.02	0.02	0.392	[−0.06; 0.02]
Behaviors ← Attitudes (γ_9_)	0.16	0.03	<0.001	[0.11; 0.22]
Behaviors ← SDM (γ_10_’)	0.04	0.03	0.221	[−0.02; 0.09]
Behaviors ← NSDM (γ_11_)	−0.03	0.02	0.209	[−0.08; 0.02]
Indirect Effect				
γ_7_ xγ_9_	0.09	0.02	<0.001	[0.06; 0.11]
Total Effect				
Behaviors ← SDM (γ_10_)	0.12	0.03	<0.001	[0.07; 0.17]
Covariance				
Positive Emotions ↔ Negative Emotions (ϕ_12_)	−0.58	0.02	<0.001	[−0.62; −0.54]
Positive Emotions ↔ GAD (ϕ_13_)	−0.25	0.02	<0.001	[−0.30; −0.20]
Negative Emotions ↔ GAD (ϕ_23_)	0.38	0.18	<0.001	[0.33; 0.42]

Notes. Bootstrapped standardized estimates *n* = 1524. *SDM* = Self-determined motivation; *NSDM* = Non-self-determined motivation. *GAD* = Generalized Anxiety.

## Data Availability

The datasets generated during and/or analyzed during the current study are available from the corresponding author upon reasonable request.

## Abbreviations

The following abbreviations are used in this manuscript:

PEB	Pro-Environmental Behaviors
SDT	Self-Determination Theory
SDM	Self-Determined Motivation
NSDM	Non-Self-Determined Motivation

## Appendix A. Data Preparation

Before conducting the analysis, the data were screened for insufficient effort responding, missing data, and data normality. The data were sequentially screened using multiple approaches to identify participants who may not have responded truthfully or attentively (i.e., Mahalanobis Distance, validation questions, and long string) (Curran, 2016; Huang et al., 2012). Out of the original sample, 340 participants were flagged as multivariate outliers at *p* < 0.001. These participants were removed from the analysis. Then, the data were screened using the three attention check questions (e.g., “Please select 7—Strongly agree to this item”). This identified an additional 440 participants who failed to properly respond to two or more validation questions. Lastly, the data were screened to identify overly invariant responses using the long string indicator (Johnson, 2005). In order to be deemed problematic, a participant had to provide, on three or more scales, a string of consecutive responses equivalent to half or more of the scale length. As a result, an additional 216 participants were flagged by the long string index on at least three scales throughout the survey. After screening, the analytical sample comprised 1527 participants who were deemed to have answered questions truthfully and attentively.

We then imputed the missing data at an item level, using single-imputation on the variables used to generate the profiles (i.e., need satisfaction, attachment anxiety, and avoidance of the mother and the father). Given that 1% of the data were missing for all the items, the data were deemed missing completely at random and suitable for imputation. Missing data were imputed using the mice package (van Buuren & Groothuis-Oudshoorn, 2011). After imputing the missing data and computing the composite scores, the descriptive statistics for each of the variables were calculated (refer to Table 1). It was found that the composite score for the attachment anxiety with the mother and the father was not normally distributed (skew = [2.09; 2.59]; kurt = [3.70; 6.78]). The variables were thus transformed using a box–cox transformation using the MASS package (Venables & Ripley, 2002). Applying the transformation decreased the kurtosis and skewness of the variables. After ensuring the normality of the distribution and applying the required transformation, the scores of the need satisfaction, attachment anxiety, and avoidance with the mother, as well as with the father, were standardized and winsorized within z = +/− 3.29.

Once the profiles were generated, the same imputation process was applied to the outcome variables (i.e., environmental self-determined motivation, non-self-determined motivation, positive emotions, negative emotions, attitudes, and generalized anxiety). The variables used to generate the latent profiles were not included in the second imputation process to avoid inflating the association between the outcomes and the profiles.
